# The Epidemiology of Fusarium Wilt of Banana

**DOI:** 10.3389/fpls.2019.01395

**Published:** 2019-12-20

**Authors:** Kenneth G. Pegg, Lindel M. Coates, Wayne T. O’Neill, David W. Turner

**Affiliations:** ^1^Ecosciences Precinct, Department of Agriculture and Fisheries, Brisbane, QLD, Australia; ^2^School of Agriculture and Environment, Faculty of Science, The University of Western Australia, Perth, WA, Australia

**Keywords:** Fusarium wilt, *Fusarium oxysporum* f. sp. *cubense*, *Musa spp.*, disease containment, infection process

## Abstract

Fusarium wilt of banana (also known as Panama disease) has been a problem in Australia since 1874. Race 1 of the pathogen (*Fusarium oxysporum* f. sp. *cubense*) is responsible for damage to ‘Lady Finger’ (AAB, Pome subgroup) and other less widely grown cultivars such as ‘Ducasse’ (Pisang Awak, ABB). Subtropical Race 4 (STR4) also affects these cultivars as well as Cavendish cultivars (AAA) in southern Queensland and northern New South Wales where cold temperature predisposition is involved. Tropical Race 4 (TR4) has led to the demise of the Cavendish industry in the Northern Territory, and its presence was confirmed in a North Queensland plantation in 2015, which warranted destruction of all banana plants on the property; as of this writing (April 2019), TR4 has spread to two adjacent properties. This review, which was commissioned by Biosecurity Queensland in response to the 2015 TR4 outbreak, considers the key epidemiological factors associated with the onset of a Fusarium wilt epidemic. Resistance to TR4, which is mediated by events following entry by the pathogen into the xylem, is not present in any commercially acceptable banana cultivar. Also, there is no effective chemical agent that can be used to manage the disease. Besides prevention, very early recognition and rapid containment of a disease outbreak are necessary to prevent epidemic development. A good understanding of the key factors responsible for disease development is required when devising practical protocols for the destruction of infected plants, treatment of surrounding infested soil, and reduction of inoculum in plant residues and soil.

## Introduction

“*Panama disease can transform a living plantation to a dead loss in a few months*” (Carefoot and Sprott, 1969).

This review of the epidemiology of Fusarium wilt of banana (Panama disease) is in response to an outbreak of the disease that was detected in a Cavendish (AAA) banana plantation at Tully (17.9329°S, 145.9236°E) in North Queensland, Australia in March 2015. Molecular and vegetative compatibility group (VCG) analyses showed that the pathogen [*Fusarium oxysporum* Schlecht. f. sp. *cubense* (E.F. Smith) Snyder and Hansen] (Foc) belonged in clonal population VCG 01213/16, colloquially known as tropical race 4 (TR4). This population, which is thought to have evolved with its banana host in the Indo-Malayan region (Ploetz and Pegg, 1999), was first detected in Australia in a Cavendish plantation near Darwin in the Northern Territory in 1997.

Foc is genetically heterogeneous and possibly has had several origins (polyphyletic). Vegetative compatibility has been used to characterize the pathogen with more than 20 VCGs being reported (Ploetz and Pegg, 1997; Ploetz, 2019). TR4 (VCG 01213/16) is just one of several distinct populations of Foc that can attack Cavendish (Pegg et al., 1993; Bentley et al., 1998; Ploetz and Pegg, 1999; Moore et al., 2001; Buddenhagen, 2009). It is much more aggressive than the so-called subtropical race 4 (STR4) strains that have been reported from Australia, Canary Islands and South Africa. STR4 strains attack Cavendish plants that have been predisposed by cold winter temperatures, soil water saturation, or drought, but are not known to attack Cavendish in the tropics. TR4 does not require predisposing factors to affect Cavendish, and susceptibility to this strain does not change with the physiological status of the host.

TR4 is a soil-borne fungus with strictly asexual reproduction, producing microconidia, macroconidia and chlamydospores as survival structures. It is extremely difficult to manage because the pathogen persists in soil or colonized host tissue by producing chlamydospores with a long survival potential, by growing as hyphae in organic residues, and by invading and surviving as asymptomatic endophytes in a range of non-host plants (Pittaway et al., 1999; Hennessy et al., 2005). In addition, the pathogen can cause disease at low inoculum levels, can reside at some depth in the soil, and the disease can have a long incubation period. Presently, there is no practical and effective way of detecting an infected plant until external symptoms are expressed.

Symptomatic plants are the basic parameter for studying the progress of the epidemic, and also for indicating the distribution of potential new inoculum that may be released into the soil. With all plant diseases there is an incubation period during which no symptoms are expressed in the host. The time between root infection and the development of Fusarium wilt symptoms can be between two to six months (Rishbeth, 1957). This long incubation period is influenced by the initial inoculum level, the susceptibility or resistance of the host plant, and prevailing environmental conditions. When symptoms are not evident it is difficult to know where and whether the pathogen is in a plantation. However, even in scarcely infested fields, infection eventually occurs as banana roots ramify though the soil, contact the fungus, and become infected. This process may take five or more years (Rishbeth and Naylor, 1957).

The ultimate solution to TR4, an acceptable resistant cultivar, is so far unavailable. Thus the application of exclusion and early quarantine measures is the only effective way of managing the disease. Once the pathogen is found in a new area, exclusion from non-infested plantations is difficult, especially if factors that influence the epidemiology and pathology of the disease are not understood. Once exclusion has failed, it is also extremely difficult to predict the extent and duration of quarantine measures that will be needed on an infested property as it will depend on the rate of disease development, the location of new disease foci, and whether banana production is continued on the site (Stover, 1962). Most of our current knowledge is derived from studies reviewed by Stover (1962) during the ‘Gros Michel’ era. The export trade (mainly in Central America) was based on this cultivar until the 1950s when it collapsed due to devastation caused by race 1. The amount of research on the epidemiology of Fusarium wilt declined significantly following the substitution of resistant Cavendish clones for susceptible ‘Gros Michel.’ The appearance of the less aggressive STR4 strains in the 1980s and 1990s did stimulate a lot of valuable research on pathogen diversity but little on the disease itself.

Little is known about the disease cycle and host pathogen interactions of TR4 (Buddenhagen, 2009); much more research is required as there are still many difficulties associated with the development and application of containment measures. A more effective method of detecting and destroying the host plant and treating the surrounding soil to disrupt the production and dispersal of initial propagules of the pathogen is a priority (Ordonez et al., 2015). In combination with established clean production methods to minimize pathogen dispersal (see the section *TR4 Control and Containment in Queensland, Australia*), this will slow down, but may not stop the spread of this threatening disease.

## History of the Disease in Australia

**Figure 1:**
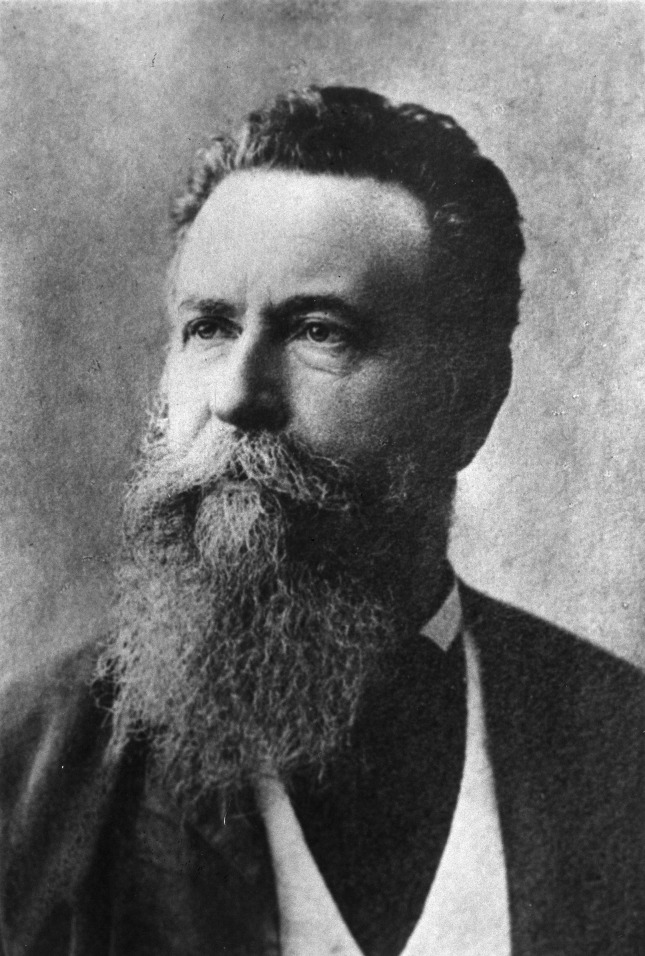
Dr Joseph Bancroft (John Oxley Library).

The first report and description of Fusarium wilt of banana in the world was from Australia. In 1874, Dr Joseph Bancroft (**Figure 1**) found banana plants (‘Sugar,’ AAB, Silk subgroup) with a fungal wilt disease at Eagle Farm (27°S 431°E) near Brisbane (Bancroft, 1876). Bancroft’s description of the symptoms leaves no doubt that he was dealing with a Fusarium wilt disease (Pegg et al., 1996). He recognized the ease by which the pathogen could be spread by vegetative propagation and advocated careful selection of disease free planting material. It is of interest to note that this was also the first recorded plant pathological investigation in Queensland. The disease was again recognized by Tryon (1912), who noted the susceptibility of ‘Sugar’ and ‘Gros Michel’ and the resistance of Cavendish.

In 1947, Magee and Simmonds independently reported the susceptibility of ‘Lady Finger’ (AAB, Pome subgroup) and recognized that this cultivar was not as susceptible as ‘Sugar’ or ‘Gros Michel’ (Magee, 1947; Simmonds, 1947). The ‘Lady Finger’ cultivar became widely grown, but by the 1960s Fusarium wilt was also recognized as a serious problem for this cultivar. The disease currently causes significant damage in ‘Lady Finger’ plantations in subtropical, eastern Australia.

Cavendish was regarded as being highly resistant to the disease, but in 1953 a small number of plants of the Cavendish cultivar ‘Williams’ were found affected by Fusarium wilt in southern Queensland (Purss, 1953). An isolate from ‘Williams’ was pathogenic to both Cavendish and ‘Lady Finger,’ but an isolate from ‘Lady Finger’ did not affect Cavendish. This was possibly the first occurrence of Fusarium wilt caused by STR4 in Australia.

The disease did not appear again in Cavendish plantations in southern Queensland until 1976 (RA Peterson, personal communication, 1976), but since only an occasional plant was affected, it was not recognized as being important. It was considered to be a break down in resistance in Cavendish due to poor soil conditions, as affected plants were growing in shallow clay soils subject to waterlogging. However, by the early 1980s many Cavendish plantations were being seriously affected in southern Queensland (Mayers, 1983) and northern New South Wales (Pegg and Langdon, 1987). Most outbreaks occurred in plantations where wilt susceptible ‘Lady Finger’ had been replaced with resistant Cavendish cultivars. These outbreaks were shown to be caused by a new strain of the fungus (Pegg and Langdon, 1987) which is now referred to as subtropical race 4 (STR4) (Ploetz et al., 1990; Ploetz, 2015). This strain was also found to be present, often with race 1 strains, in ‘Lady Finger’ plantations.

In 1992 Fusarium wilt was detected in 10 out of 153 Cavendish plantations at Carnarvon in Western Australia. A race 1 strain of the pathogen was involved and adverse environmental conditions (flooding and drought) were implicated in the disease outbreak. This strain of Foc was thought to have been introduced with windbreak banana plants (an edible diploid of *Musa acuminata*), which were imported directly from Java and Singapore before 1930 (Pegg et al., 1995; Shivas et al., 1995).

In 1997, an outbreak of Fusarium wilt occurred on Cavendish in the Northern Territory. This was the first detection of TR4 in Australia (Conde and Pitkethley, 2001). It has caused significant damage and a dramatic decline in commercial production of bananas in the Northern Territory. In March 2015, TR4 was detected in Australia’s major banana production region near Tully in North Queensland (O’Neill et al., 2016) where it has created a landscape of fear.

## Infection and Disease Progression

Fusarium wilt of banana is a classical vascular wilt disease. The pathogen is considered to be a hemibiotroph, since the initial infection establishes a biotrophic relationship with the host but eventually transitions to a necrotroph where host tissue is killed (Dita et al., 2018; Ploetz, 2019). Multiple cycles of infection occur in a banana plantation that is infested with Foc (Ploetz, 2015). With the exception of sucker rhizomes being infected directly *via* vascular connection with diseased parent plants, all infections originate from secondary and tertiary roots (Wardlaw, 1961). The larger roots are rarely infected directly, and while the plant can occasionally be infected through the rhizome or pseudostem, such infections usually remain localized. When surface wounds or weevil borer (*Cosmopolites sordidus*) tunnels are invaded by the pathogen, there is some hyphal invasion of surrounding cells (Ploetz and Pegg, 1999). However, deeper invasion of rhizome tissue or leaf bases is prevented by the rapid formation of suberized protective barriers by the host plant. When a sucker is severed from the parent plant, there are numerous exposed injured vessels. These vessels rarely become infected, and if they do become infected, the pathogen rarely penetrates more than one centimeter before a protective barrier is produced by the host plant (Wardlaw, 1961).

**Figure 2:**
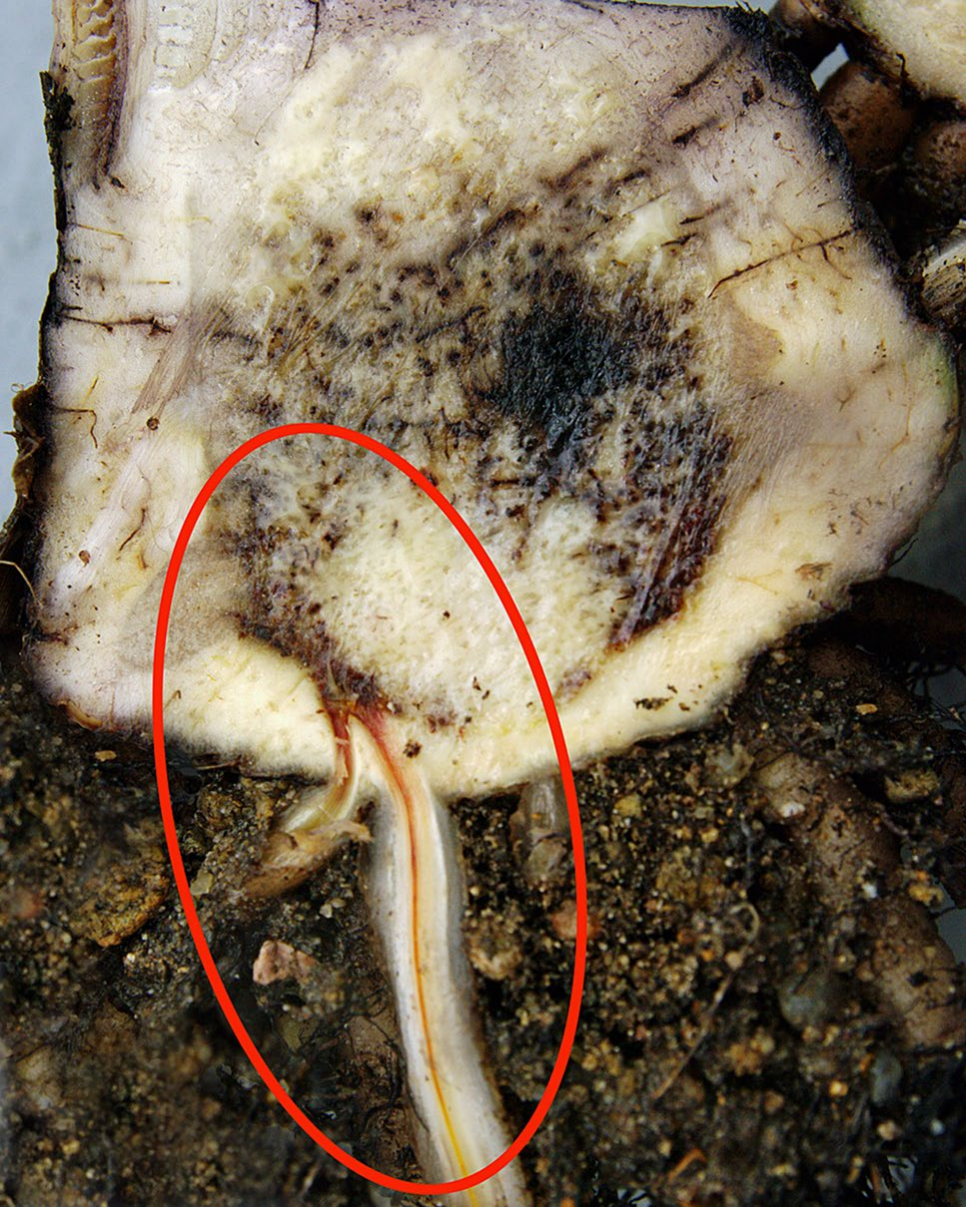
Dark brown discoloration of vascular tissue in a banana root caused by Foc. (W. O’Neill).

**Figure 3:**
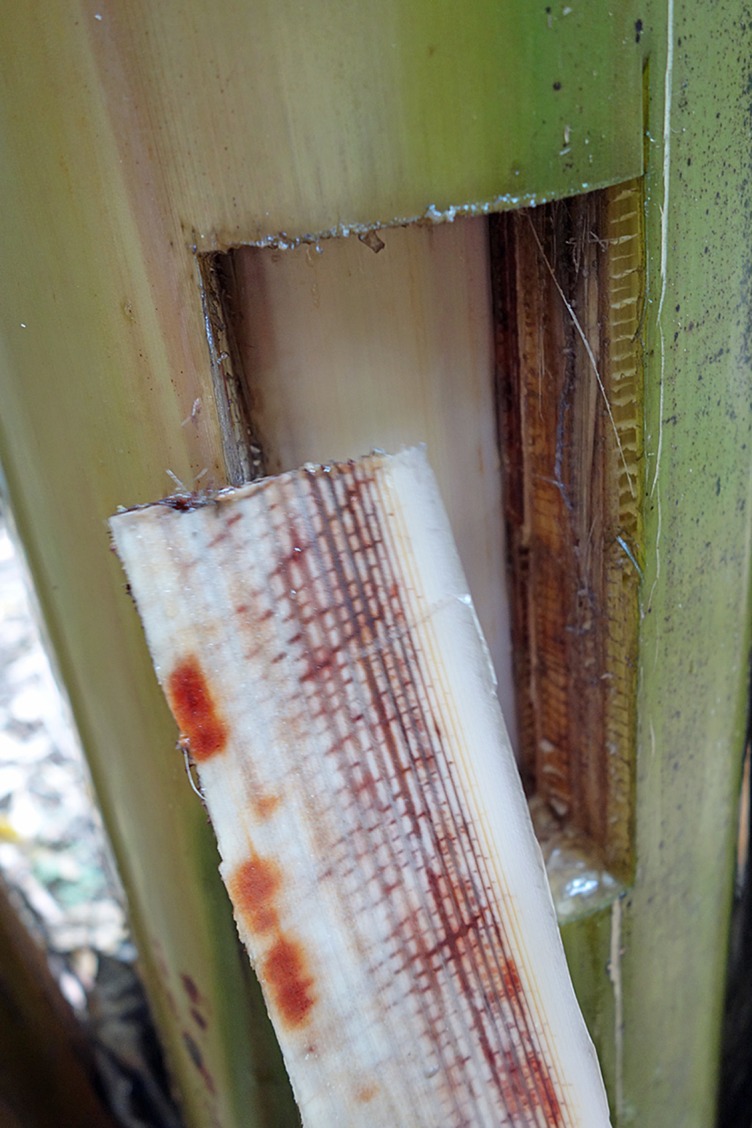
Vascular discoloration in xylem vessels (W. O’Neill).

Chlamydospores in the soil are stimulated to germinate by nutrients in the exudates from banana roots and non-hosts, or contact with pieces of non-colonized plant residues (Ploetz and Pegg, 1999; Dita et al., 2018). Those infecting the tips of secondary and tertiary roots of banana penetrate the root cap and zone of elongation and establish an intercellular parasitic relationship in the root cortex, before entering xylem vascular elements (**Figure 2**). The pathogen gains its nutrients from cell exudates rather than from the cell wall or the cytoplasm. The pathogen may also infect through natural wound sites along the roots. Living xylem is present in the banana root close to the apex, and as xylem becomes mature the vessels become vacuolated and the protoplasm and nucleus disappears from such elements. The living xylem is a barrier against the advance of the pathogen and the probability that the infection may reach the rhizome is low, estimated to be about one root in 20 by Rishbeth (1957). However the pathogen can move passively in the empty lumen of mature vessels (Trujillo, 1963). Initial movement through the roots can be slow, requiring some four weeks to advance 75 cm, but in mature xylem vessels, the pathogen can advance in surges of 30 cm in two to three days, with every new generation of spores. Once reaching the rhizome the pathogen can become distributed within the pseudostem in less than two weeks (MacHardy and Beckman, 1981) (**Figure 3**). Studies by Beckman et al. (1961) and Trujillo (1963) suggested that this colonization is facilitated by the extensive formation of conidia within the xylem elements, and that these spores move freely in the transpiration stream until they are temporarily blocked by the perforation plates at the end of the xylem vessels.

Once impeded by the perforation plate, the microconidia reportedly germinate and germ tubes grow through the plates and again sporulate to produce more conidia. The sequence of trapping, germination, penetration and sporulation would allow the host to be colonized quite quickly. Wardlaw (1961) however reported slender, unbranched, hyphae growing in the vascular elements of ‘Gros Michel.’ Trujillo (1963) suggested that movement of the pathogen in the plant was too rapid to be explained by growth of mycelium, noting that Foc migrated from the rhizome to the top of a 12 m-tall plant in less than two weeks. However, Cook (1981) suggested that, although less efficient, banana plants may also be systemically infected *via* hyphal extension. In more recent studies of young glasshouse plants inoculated with GFP (green fluorescent protein)-transformed STR4, Warman and Aitken (2018) sometimes observed microconidia prior to any signs of hyphae, whereas at other times observed single or multiple strands of hyphae without the presence of any microconidia in the vascular elements of the pseudostem. Based on the studies conducted to date, it would appear that both microconidia and hyphae are present in the xylem of infected plants, but the relative importance of each in the infection process is yet to be precisely clarified. Although less efficient, spread within the xylem *via* hyphal growth should not be dismissed. The extent to which this occurs, and whether this method of host colonization depends on the banana cultivar and the identity of the pathogen should be investigated.

**Figure 4:**
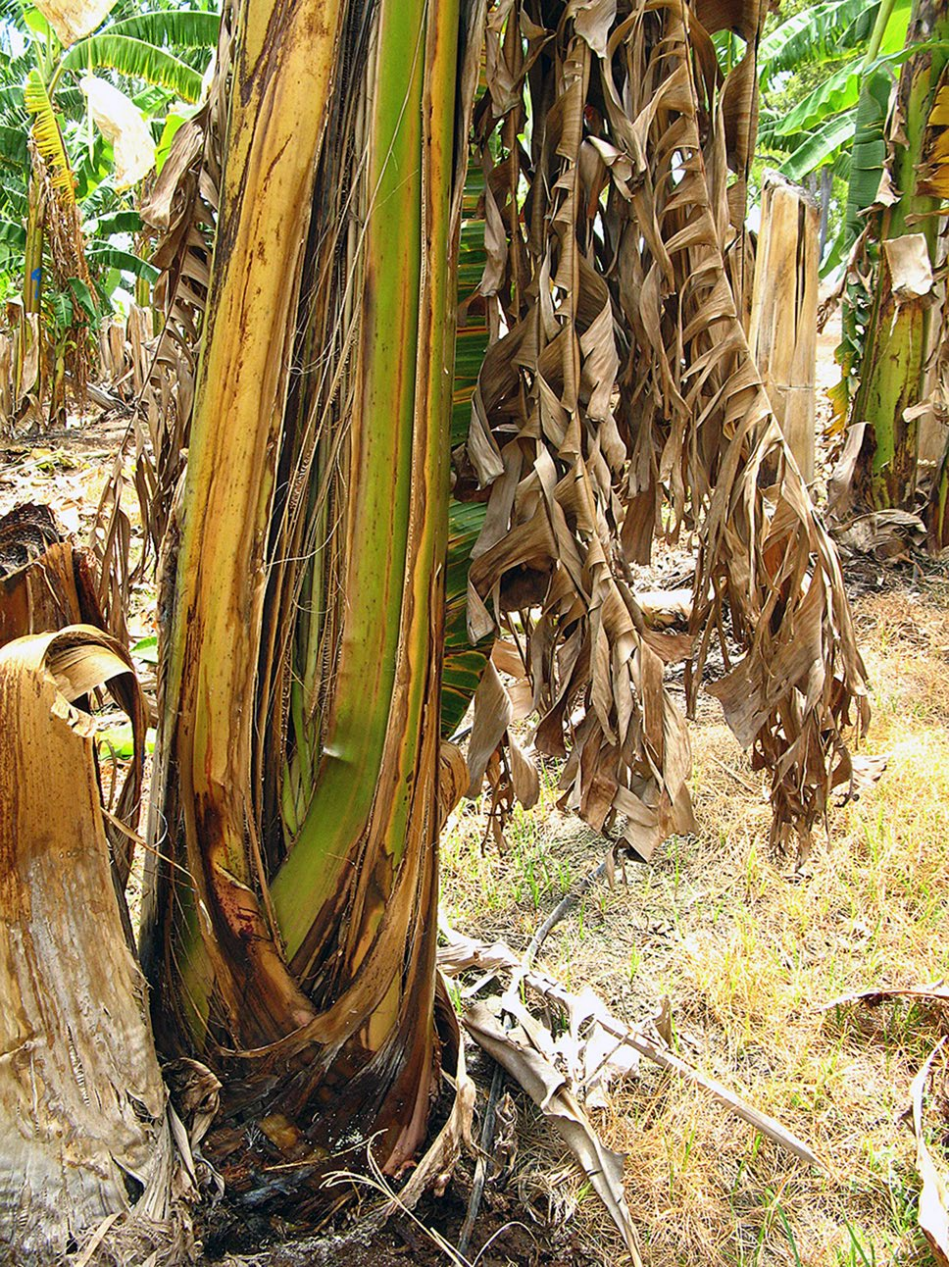
External symptoms of Fusarium wilt in ‘Lady Finger’ banana: pseudostem splitting of leaf bases (W. O’Neill).

Infections trigger a host defense response involving gels, tyloses and lignification that leads to vascular occlusion. This response is present even in susceptible cultivars (Ploetz and Pegg, 1999). In addition to trapping by the perforation plates, the pathogen can be immobilized by gels forming on the upper side of these plates. These gels will not persist unless there are other host reactions in the xylem elements, and therefore only impede colonization for a short time. If the gel persists long enough to allow tyloses to form, and then become impregnated by phenolic materials, the pathogen can be successfully contained. In resistant bananas infection is checked by the rapid deployment of these host defenses in their rootlets, roots or at the root base (MacHardy and Beckman, 1981). In susceptible banana plants, after the pathogen has systemically invaded the xylem vessel elements with appreciable invasion of the rhizome (Simmonds, 1966), a severe water shortage develops due to vascular plugging. This impaired water movement leads to reduced transpiration and the expression of external symptoms. A common initial symptom is the appearance of a faint pale yellow streak at the base of the petiole of the oldest leaf. This is followed by leaf chlorosis which progresses from lower to upper leaves, wilting of leaves and longitudinal splitting of their bases. Splitting is more common in young, rapidly growing plants (Stover, 1962) (**Figure 4**). Systemic invasion of the xylem vessels in the pseudostem does not necessarily need to occur for wilt symptoms to become apparent (Moore, 1995). The involvement of toxic metabolites in pathogenesis has been proposed. Fusaric acid which is produced by Foc and other *Fusarium* species is believed to contribute to symptom expression (Dong et al., 2014). The banana plant may be slow to show external symptoms because it has a vascular capacity two to three times its need for growth and reproduction (Beckman, 1990).

MacHardy and Beckman (1981) comment on the weak ability of Foc to invade living plant tissues. They describe how the fungus traverses the energy-rich cells of the root cortex to colonize the nutrient-poor, rather inhospitable xylem vessels encased in lignified walls. The xylem fluid contains only water which is low in carbohydrates, amino acids and minerals, has a low oxygen concentration and a fluctuating pH. Perhaps through its prolonged coevolution with its banana host, it has developed the ability to adapt to the isolated xylem environment where there is no microbial competition and where it can move freely with help from the transpiration stream to complete its life cycle. The transpiration stream will also deliver nutrients to the hyphae. Late in pathogenesis, when the vascular tissue has been fully colonized, the pathogen escapes from the xylem into the adjacent parenchyma and cortex to invade plant tissue weakened by water deficit. Chlamydospores and conidia are then produced in the degraded host tissue, and are released into the environment when these tissues collapse.

A number of recent studies have used GFP-transformed isolates of Foc to visualize the movement of the pathogen through the banana plant. Despite variation in the Foc strains, banana cultivars, plant age at inoculation and inoculum levels used in the individual studies, all demonstrated a similar pattern of root infection whereby the fungus directly penetrates the epidermal cells of the root tip followed by intercellular growth along the elongation zone (Xiao et al., 2013; Li et al., 2017; Warman and Aitken, 2018). By 10 days after inoculation with GFP-transformed Foc, the pathogen was found inside the xylem tissue of roots in Cavendish cv. B.F. plants (Xiao et al., 2013) and Cavendish cv. Williams (Warman and Aitken, 2018). Colonization of the roots, rhizome and lower pseudostem tissue of ‘Lady Finger’ plants by STR4 was markedly slower than in Cavendish plants (Warman and Aitken, 2018). Hyphae were confined to the xylem vessels of both Cavendish and ‘Lady Finger’ plants while the leaf sheaths were healthy and intact, but were observed in the gas spaces of leaf sheaths once the outer leaf sheaths and leaves began to senesce. At a more advanced stage of disease development, hyphae and sporodochia were observed protruding from stomata in the leaf sheaths. Production of chlamydospores also occurred, both internally within the gas chambers and externally on the outer surface of leaf sheaths. This suggests that senescent leaf sheaths are a significant source of Foc inoculum, and that cultural practices such as de-leafing may increase the risk of returning chlamydospores to the soil.

## The Influence of Climatic and Soil Factors on Disease Development

Many factors affect Fusarium wilt of banana under field conditions, most importantly the susceptibility of the banana cultivar. Plant development stage is also significant, as mature plants are more resistant than younger plants (Brake, 1990). However, weather events (prolonged wet or dry conditions, extremes in temperatures, storm damage) and soil conditions (poor soil drainage and aeration, unfavorable chemical or physical conditions) also have a major influence on the disease (Brake et al., 1995). Many of these factors directly impact the host plant and its response to the pathogen, whereas others have a direct effect on the pathogen.

### Weather Events

Weather affects the incidence and severity of Fusarium wilt during infection, systemic infection of the xylem, and the development of wilt symptoms (Cook, 1981). Growth and survival of the pathogen in the root zone are favored by dry soil conditions under which the fungus is still able to extract sufficient water for its own growth and reproduction but is less likely to be antagonized by or compete with other microorganisms. After infection, disease development will depend on sufficient water being available for pathogen growth and dispersion in the xylem fluid. An internal water deficit caused by dry conditions or waterlogging promotes symptom expression (Peng et al., 1999; Pattison et al., 2014).

#### Rainfall

In Puerto Rico, Fawcett (1913) noted that shortly after the wet season began the incidence of symptomatic plants increased. Rishbeth (1957) found that wilt incidence was greatest when conditions were most favorable for plant growth. He observed that there was a slow but steady appearance of diseased plants during dry conditions, but that the incidence of wilted plants increased four-fold following two months of heavy rainfall. Stover (1962), reported that the disease developed more slowly on ‘Gros Michel’ in Honduras in the dry season, and that wilt incidence was highest when rainfall and temperatures favored maximum plant growth. Simmonds (1966) suggested that drought depressed, and heavy rain favored the development of, the disease. He also indicated that an actively growing plant favored disease development.

**Figure 5:**
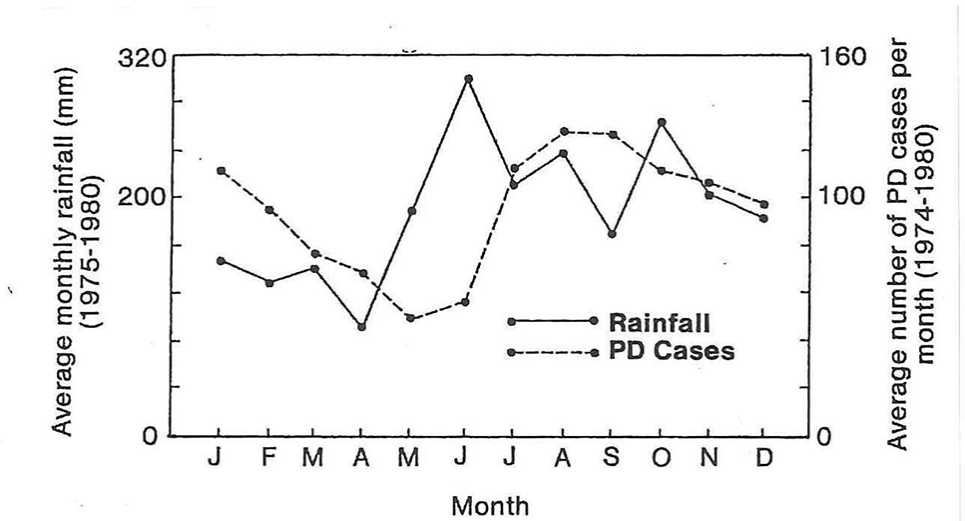
The average monthly rainfall and number of Fusarium wilt cases per month show increased disease incidence peaking at three months after high rainfall (from Epp, 1987).

Epp (1987) studied the epidemiology of Fusarium wilt in Cavendish type banana ‘Umalag’ for 4 years in the Philippines. He showed that symptoms developed after rain events, and that there was a correlation between heavy rains and increased disease incidence (**Figure 5**). He also noted that 95% of the symptomatic plants had bunched or were about to bunch; the disease was rarely found on Cavendish plants less than six months old, but even young plants of ‘Gros Michel’ succumbed, presumably due to their greater susceptibility. It should be noted that VCG 0122 (a far less aggressive population of the pathogen), rather than VCG 01213/16 (TR4), was probably involved in these studies, as VCG 01213/16 was not confirmed in Mindanao for another decade. Epp reports that he was able to control the disease by the early and accurate identification of infected plants, creation of adequate buffer zones around infected mats, good weed control and optimal nutrition. The successful management of Fusarium wilt using such interventions would be highly unlikely to have been possible in the presence of TR4 as it is much more aggressive than other known populations (Ploetz, 2019).

#### Temperature

Temperature is a critical factor in wilt development (Rishbeth, 1957). Peng et al. (1999) noted that the growth of Fusarium wilt pathogens is usually greatest at 28°C, and inhibited above 33°C and below 17°C. Stover (1962) also noted that plant growth was reduced and disease development was slow during winter, possibly due to reduced transpirational stress on the plant. It has also been proposed that cold winter temperatures in the subtropics predispose Cavendish to systemic infection by STR4 (Moore, 1995). The optimum temperature for growth of Cavendish is between 22 and 31°C (Turner and Lahav, 1983; Robinson 1990). The assimilation of carbon dioxide and rate of leaf emergence decrease when the temperature falls below 22°C, with growth ceasing at 14°C. At low temperatures, light bleaches chlorophyll, primary root growth stops, and tertiary roots die without the formation of new ones; thus, the uptake of water and nutrients is severely affected.

Moore (1995) studied the effect of winter temperatures on susceptible (‘Williams’) and resistant (‘Dwarf Parfitt’ Cavendish plants. In August, ‘Dwarf Parfitt’ had a higher F_v_/F_m_ ratio (chlorophyll fluorescence induction) than ‘Williams,’ indicating that it sustained less damage due to winter chilling (**Table 1**). ‘Dwarf Parfitt’ maintained leaf area during winter, while that of ‘Williams’ declined. Even though ‘Dwarf Parfitt’ had a lower CO_2_ assimilation efficiency per kg dry weight of total biomass than ‘Williams’ plants at the end of autumn, it maintained a higher efficiency than ‘Williams’ during cold weather.

**Table 1:** Chlorophyll fluorescence induction F_v_/F_m_ ratio of Cavendish in winter and spring at Wamuran, Queensland (27.0359° S, 152.8627° E). Data are mean values of single leaves of three plants of each cultivar ± standard errors. From Moore (1995).

Chlorophyll Fluorescence Induction (F_v_/F_m_ ratio)		
Cultivar/genotype	F_v_/F_m_ ratio	
	Winter (August)	Spring (October)
Dwarf Parfitt	0.520 ± 0.038	0.722 ± 0.013
Williams	0.354 ± 0.061	0.653 ± 0.018

At both 20 and 28°C, Brake et al. (1995) reported severe symptom development in plants inoculated with race 1 or STR4. In a glasshouse experiment, Peng et al. (1999) found that disease severity increased as temperature increased from 24 to 34°C. At 14°C (a temperature unfavorable for the growth of banana) symptoms did not develop but the pathogen could be isolated from the plants.

#### Cyclonic Weather

Rishbeth (1957) found that disease increased markedly following cyclonic weather. He attributed this to the production of many highly susceptible new roots by storm damaged plants. He had previously demonstrated that young roots of ‘Gros Michel’ were more susceptible to infection than older roots. Any catastrophic event that causes mechanical breakage of the root system will rapidly increase disease incidence and severity, especially if flooding or waterlogging occurs following the storm event.

### Soil Factors

#### Influence of Soil Type

Stover (1956) suggested that some soil factors weaken plant resistance and play a vital role in disease development. He indicated that the disease is more serious in light sandy soils than in heavy clay soils. This is probably due to the effect of the sandy soil on the water relations of the host plant, but also *Fusarium* species are strongly aerobic and are favored by soil water contents of less than field capacity. Presumably there would also be less competition or antagonism from other soil microorganisms in the sandy soil.

#### Suppressive Soils

There are some banana soils with chemical, physical and microbiological properties that suppress disease development. These are referred to as suppressive soils, as opposed to conducive soils in which the disease progresses unrestrained on susceptible banana clones. Suppressive banana soils have been reported in Central and South America, Australia, Canary Islands and South Africa for races other than TR4. Even with TR4, one would expect some variation in the incidence and severity of the disease over time in different production areas.

Stover (1962) summarized studies on the impact of soil type on the development of Fusarium wilt on ‘Gros Michel,’ where different soils in Central America were initially categorized as ‘non-resistant,’ ‘semi-resistant,’ and ‘resistant,’ based on the rate of disease spread; they were later referred to as ‘short-life’ or ‘long-life’ soils. Reinking and Manns (1933) also described suppressive soils in Central America. Stotzky and Martin (1963) correlated suppressiveness with the presence of montmorillonoid clay in soils in Central America and Ecuador; they suggested that suppressiveness was associated with greater biological activity in these soils.

Although suppressive soils often have similar abiotic properties (higher pH, higher organic carbon content, clay texture), it is generally accepted that suppression is mainly through the impact of these properties on the saprophytic soil microflora. Soil microorganisms play a key role in suppressing soil-borne diseases, mainly *via* antagonism or competition (Cook and Baker, 1983; Alabouvette, 1986).

Control of Fusarium wilt in susceptible cultivars has not been achieved using practical biological or cultural methods. However, the existence of soils where *Fusarium* is naturally suppressed does indicate that such control may be possible with a better understanding of the physical and biological environment, pathogen activity and host susceptibility. Peng et al. (1999) observed differences in disease expression in a Cavendish planting at Carnarvon (25°S, 114°E) in Western Australia and found differences in chlamydospore germination and disease severity in laboratory and glasshouse tests using soil from areas where there were no wilted plants and soil nearby where disease was clearly evident. The suppressive soil had a higher population of actinomycetes and bacteria than the conducive soil, and the potassium concentration in the suppressive soil was 3.5 times higher.

In a study of ‘Pisang Awak’ and race 1, Pattison et al. (2014) demonstrated that symptom incidence and severity of Fusarium wilt was reduced with a ground cover of Pinto peanut (*Arachis pintoi*) that reduced water stress and enhanced soil microbial activity and diversity. Disease reduction was significant, but not great (20% compared to the non-ground cover treatment). Although it was not tested against TR4, the authors suggested that ground cover could reduce its impact in Cavendish somaclones which are not completely resistant to Fusarium wilt, but currently no such information is available.

#### Soil Water Content and Aeration

Both Rishbeth (1957) and Stover (1962) reported a higher incidence of wilt disease in poorly drained soil, when there was temporary flooding of the root zone. Stover (1962) found reduced activity of *Fusarium* in wet soils and attributed this to elevated levels of carbon dioxide which favor chlamydospore germination and hyphal development, but inhibit the formation of new chlamydospores, thus decreasing the population of the pathogen. However, he also suggested that water-saturated, oxygen-deficient conditions predispose the host to infection. Turner et al. (2007) noted that banana growth and productivity are negatively impacted by poor drainage due to low oxygen content. The first part of the banana root to die as oxygen is reduced in soil is the root tip. Roots begin to die if soil is waterlogged for more than six hours. Aguilar et al. (2000) found that soil flooding and the resultant hypoxia or anoxia greatly restrict oxygen to banana roots, which made them more susceptible to infection.

#### The Influence of Nematodes on **Fusarium** Wilt

In some plants, nematodes predispose the host to infection by Fusarium wilt pathogens. For example, the interaction between the root-knot nematode *Meloidogyne incognita* and strains of *Fusarium oxysporum* f. sp. *vasinfectum* causing Fusarium wilt of cotton in the USA is probably the most widely recognized disease complex in the world (Smith et al., 1981; Colyer, 2007).

*Fusarium oxysporum* was a common inhabitant of root lesions caused by the burrowing nematode (*Radopholus similis*), although isolates recovered by Blake (1961) were unable to invade the host vascular system. On Cavendish in the Philippines, Epp (1987) found no association between nematode infestations (*Radopholus*, *Meloidogyne*) and the incidence or severity of Fusarium wilt. However, with ‘Gros Michel,’ Loos (1959) reported that *R. similis* aggravated disease expression and reduced the time between inoculation with Foc and symptom expression, even though its presence was not necessary for symptoms to develop.

**Figure 6:**
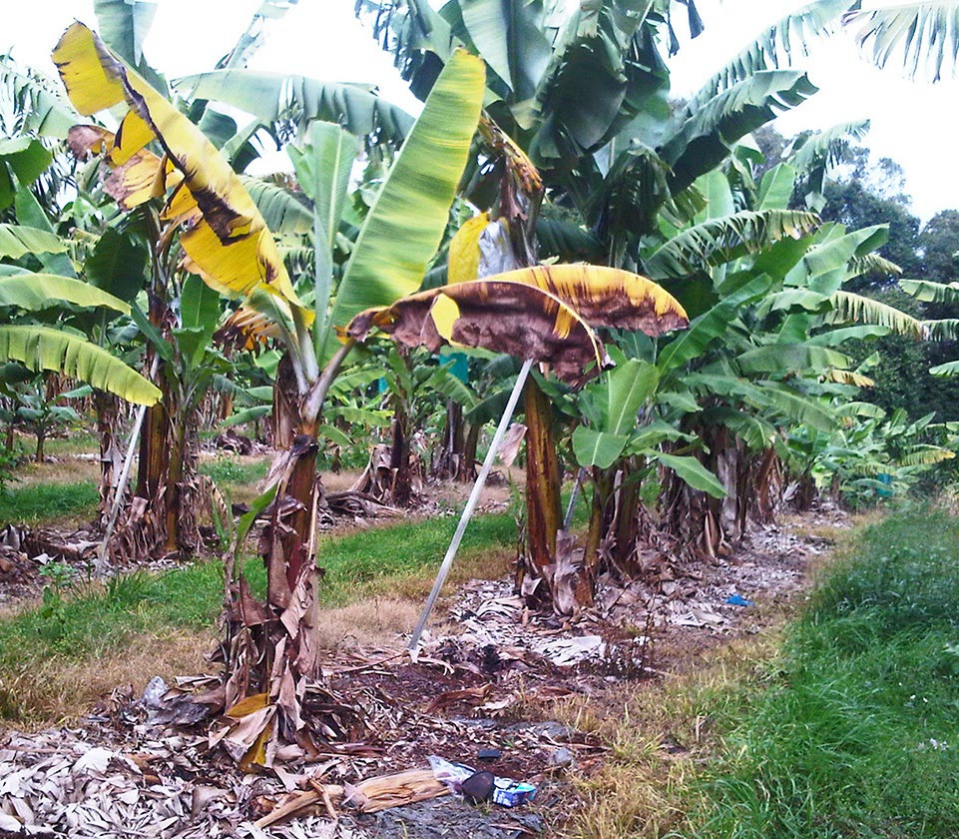
Cavendish plant affected by Race 1 Foc (D. Peasley).

In 2010–2011, Cavendish plants growing in subtropical New South Wales succumbed to a race 1 clonal population (VCG 0124) (**Figure 6**). The plants were heavily infested with both *R. similis* and *Helicotylenchus multicinctus*, but Fusarium wilt symptoms did not develop in Cavendish in a glasshouse pathogenicity test with the isolate, alone or in combination with two different inoculation rates of *R. similis*.

#### Chemical Factors

Reports on the role of chemical factors on disease development, especially fertilizer applications and soil pH, are often contradictory. This can be attributed in part to the difficulty in separating their effects on the host from those on the pathogen.

Fusarium wilt diseases in other crops are usually most severe in sandy soils with a low pH. Thus, the adjustment of pH to near neutrality by liming is used to inhibit wilt development in a range of crops. Disease suppressive banana soils in Jamaica and Central America had a neutral or slightly alkaline pH (Stover, 1962). Reducing the impact of Fusarium wilt diseases by liming soils may in part be due to limiting the availability of micronutrients to the fungus, as well as enhancing the activity of a competing microbial flora (Jones and Woltz, 1970). It is interesting to note that Peng et al. (1999), in pot experiments with conducive and suppressive soils from Carnarvon, Western Australia, found the reverse effect of pH. Disease developed more slowly in acid soils than in alkaline soils (pH 8) and they attributed this to the influence of pH on chlamydospore germination, as high pH favored chlamydospore germination. A survey by Turner et al. (1989) included one plantation at Carnarvon that had soil with a pH of 8.7 and a measured annual yield of 38 t/ha. This indicated that under the growing conditions and soils at Carnarvon, WA, bananas can be productive at a high pH, so it was important for Peng et al. (1999) to investigate the behavior of Foc with treatments in the high pH range.

Mineral nutrients that influence wilt development include nitrogen (N), potassium (K), phosphorus (P), calcium (Ca), magnesium (Mg), zinc (Zn), iron (Fe), and manganese (Mn) (Datnoff et al., 2007). Although none of these alone would be useful in reducing disease incidence or severity, maintaining a correct nutritional balance is important for plant health and prolonging the life of the plantation (Rishbeth, 1957).

Although N affects many plant diseases, its form rather than its total amount is often most important in these interactions (Huber and Watson, 1974). Regarding Fusarium wilts, nitrate (NO_3_^−^) reduces, whereas ammonium (NH_4_^+^) increases, their severity. For example, Simmonds (1966) noted that applying excessive ammonium sulfate was the most effective way of encouraging Fusarium wilt. Epp (1987) found that NO_3_^−^ increased the time between the initial expression of symptoms and death of the plant compared to NH_4_^+^; regardless, all plants died regardless of the nitrogen source.

Brown et al. (1997) stated that ammonium nitrogen lowers the pH in the rhizosphere, as hydrogen ions are released into the soil as a result of nitrification. High rates of urea are used to treat infested soil during destruction and containment procedures. Ammonia (NH_3_), produced by enzymic hydrolysis of urea, and nitrite (NO_2_^−^/HNO_2_), produced during subsequent nitrification, are directly toxic to *Fusarium* propagules (Sequeira, 1963). Ammonia accumulation also occurs when high amounts of some forms of organic matter (e.g. chicken manure) are incorporated into soils, and this can lead to disease suppression through toxicity to the pathogen and by increasing the population of microbial antagonists.

“Other than nitrogen, calcium nutrition is perhaps the most important nutritional factor for management of diseases” (Rahman and Punja, 2007). Nevertheless, there are few examples that document the impact of calcium on a Fusarium wilt. Peng et al. (1999) added calcium carbonate, calcium hydroxide, calcium sulfate and iron chelates to soils in pot experiments and reduced disease severity and chlamydospore germination of Foc by one-third to one-half. Although it was unclear how/whether the calcium materials affected the development of Fusarium wilt, it was suggested that the iron chelate acted by reducing the availability of iron that is necessary for chlamydospore germination. There is a need to take such studies to the field to see what happens in the natural world.

Although the effect of K on disease development cannot be generalized, it can reduce the severity of plant diseases (Datnoff et al., 2007). Rishbeth (1957) indicated that soil potassium levels and Fusarium wilt incidence were inversely correlated, and that the highest correlations were where potassium levels were 3.3 times higher than surrounding areas. Similarly, a putatively suppressive soil at Carnarvon in Western Australia had 3.5 times more K than conducive soil (Peng et al., 1999).

Zinc is the most important minor element in bananas. Fernandez-Falcon et al. (2004) reported that wilt was more severe in two-month-old ‘Dwarf Cavendish’ plantlets with zinc deficiency. They indicated that there was relationship between zinc concentration and IAA levels, and the timely formation of tyloses. Tylose formation in the xylem is stimulated by IAA.

Stover (1990) reviewed studies in the Canary Islands where ‘healthy’ and ‘diseased’ soils were identified using chemical and physical parameters. The ‘healthy’ soils had good structure, permeability and drainage, high levels of organic matter, zinc, calcium and magnesium. Suppressive soils in Jamaica and Central America had very high phosphate and potassium levels (Simmonds, 1966). It is evident that highly fertile soils with good physical structure, and balanced fertilization will prolong the life of an infested plantation.

Although the potential for manipulating chemical factors to manage Fusarium wilt in banana has not been fully exploited, it is improbable that this disease could be fully controlled by altering soil pH or adding a specific nutrient. The complex soil environment makes this a most difficult goal, as it complicates the host × pathogen interaction and impacts how it and soil chemistry ultimately interact.

## Pathogen Spread

Pathogen spread is either passive or active. Active spread (spread of an existing infection) depends on banana roots growing to the inoculum, whereas with passive spread (spread to new areas) the inoculum is carried to the roots.

The only means of active spread of the pathogen through the soil is from plant to plant by root proximity. The fungus is present in some of the major roots of an infected plant, and as the plant dies, these roots decay and spores are released into the soil. The short-lived secondary and tertiary roots are more likely to be infected than the major roots, and they will release a small but constant supply of inoculum into the soil while the plant is still growing (Stover, 1962). Roots of adjacent healthy banana plants will grow into the root zone of the diseased plants thus accounting for mat-to-mat spread.

Banana roots arise in groups of two to four from primordia at the inner edge of the cortex of the rhizome. These primary roots (main, cord) give rise to secondary and tertiary roots. Primary roots may be several meters long, secondary roots less than 1 m, and tertiary roots, which only remain functional for about three weeks, are several centimeters long. The primary roots produce the framework for the root system and spread well out from the plant. The secondary and tertiary roots explore the volume of soil near the primary, supporting root (Turner et al., 2007), and a small undetectable inoculum level in this soil can lead to serious disease. A single propagule entering the vascular system can multiply greatly inside the plant (Nash-Smith, 1970).

Active spread can be limited by quarantine of the affected plants and their neighbors. However, once passive spread is involved the situation changes dramatically and managing spread becomes much more difficult.

**Figure 7:**
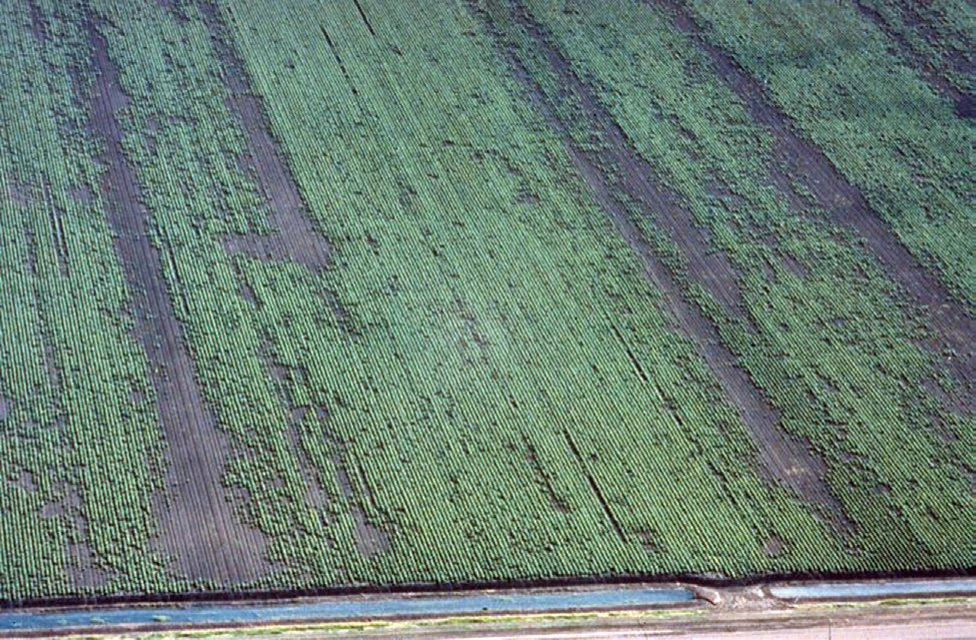
Aerial view of a cotton field affected by Fusarium wilt (*Fusarium oxysporum* f. sp. *vasinfectum*) showing spread along rows with flood irrigation water (J. Kochman)

**Figure 8:**
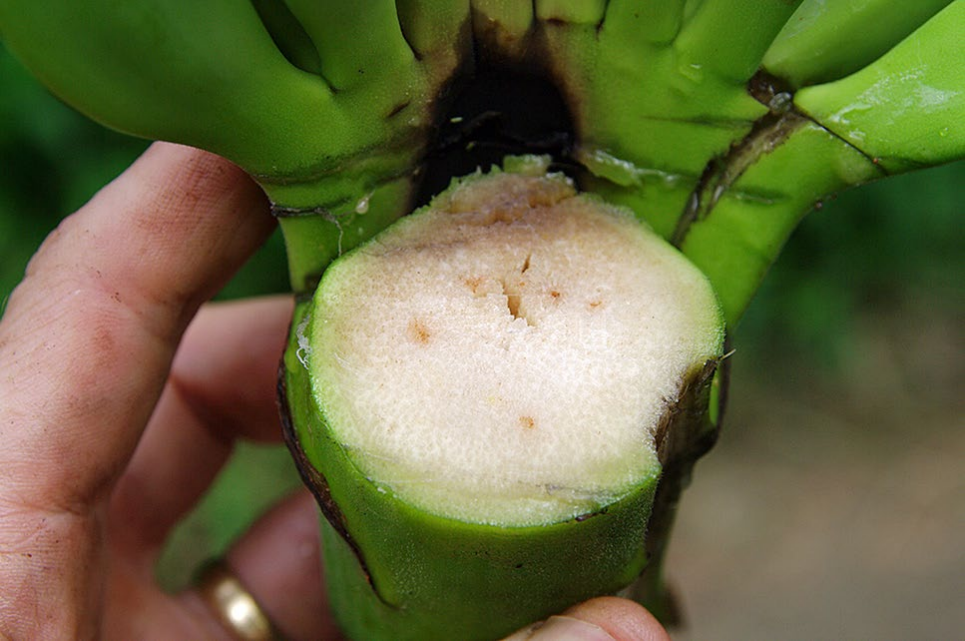
Vascular discoloration in Foc infected bunch stalk (W. O’Neill).

**Figure 9:**
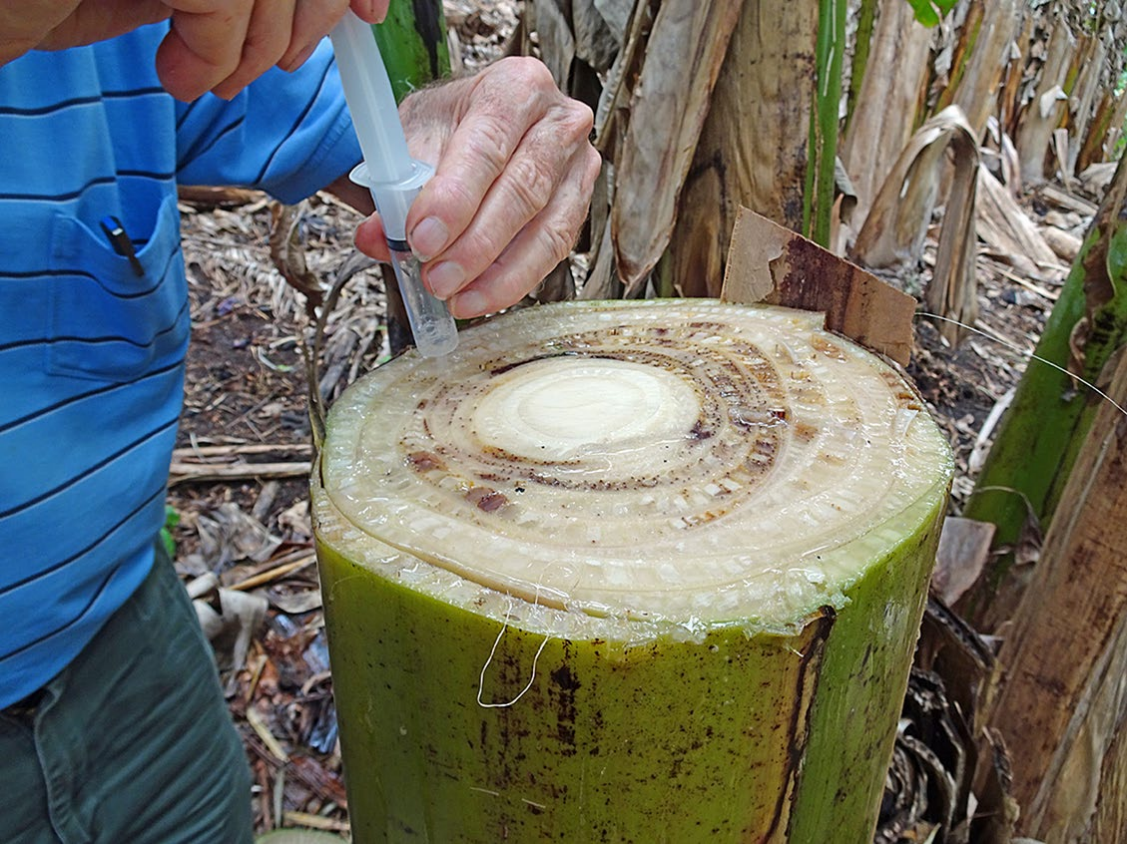
Collection of laticifer fluid and xylem sap from an infected plant for epidemiology studies (W. O’Neill).

Passive spread may occur in the following ways:

– Movement of the pathogen in water can be significant. Outbreaks of the disease commonly occur in association with irrigation and/or flooding (Stover, 1962; Su et al., 1986) (**Figure 7**). When plantations have been irrigated from contaminated dams or rivers, and when flood waters from infested land have inundated alluvial river valleys, propagules of the pathogen and infested organic residues can move considerable distances. The chlamydospores will survive better in a running river than in dam water due to superior aeration. Stover (1962) found that a large percentage of chlamydospores die after 40 days of submersion in still water, such as in dams. They survive for much longer in oxygenated water, such as in rivers. He also noted that Foc can tolerate lack of oxygen in soil for a long period, but suggested that it was lack of free oxygen plus submerged soil conditions that are the destructive factors in dams. Stover found that submerged fresh pseudostems were destroyed in 2–3 months as a result of anaerobic decay.
– If a dam is located immediately below an infested plantation, inflowing water becomes a constant threat as a source of inoculum in the dam water. As most spores sink in a day or two, it is best to take irrigation water from the surface layers of the dam and not irrigate until at least 2 days after surface run-off (from a rain event) into the dam (Deacon, 1984). Rattink (1990) also showed that microconidia of the cyclamen vascular pathogen *Fusarium oxysporum* f. sp. *cyclaminis* settled to the bottom of water containers within 24 h. This further indicates that water should not be pumped from the bottom of the dam. An original Queensland Banana Industry Protection Board recommendation for dealing with Fusarium wilt states that “Irrigation water should be taken from areas which are not contaminated by surface run-off from diseased plantations. A flotation inlet for the irrigation system should be used because fungal spores are heavy and most will sink after a day or two” (BIPB, 1989). Regardless of what precautions may be employed, irrigating from a potentially contaminated water supply is always a very risky practice.
– The pathogen can be moved in infested soil by both humans (farm machinery, vehicles, shoes and clothing of farm staff) and animals (movement greater on sticky clay soils).
– Asymptomatic planting material, either infected with or contaminated by the pathogen, is probably the most effective means of local, national and international dispersal (Stover, 1962). Su et al. (1986) found that 30–40% of suckers taken from a diseased plantation in Taiwan were infected even though they were free of symptoms, and symptoms may develop up to two years after planting such material (Rishbeth, 1957; Deacon, 1984). Tissue cultured plantlets grown under aseptic conditions in accredited nurseries with high standards of hygiene are the most reliable source of disease-free planting material.
– Infested dust and infected dry leaf debris can disperse the pathogen, as chlamydospores survive for months under dry conditions.
– The pathogen infects banana leaves, which are often used to transport fruit, as well as banana bunch stalks (**Figure 8**), which are often put back into the plantation (Deacon, 1984). Infected leaves and stalks can both initiate new disease foci.
– In infected banana fibers and other pieces of banana tissue that cling to the blade of the cane knife (machete) (Rishbeth, 1955). Rishbeth (1955) found that a machete used to cut the pseudostem of an infected plant carried 3,000 viable conidia, and that a single drop of sap contained the same number of microconidia. The sap can dribble from the machete blade and contaminate the soil. He also suggested that the spores in the sap on the blade will remain viable for several days and be a potent source of inoculum for other plantings. Wardlaw (1961), quoting Stover (1954, 1956), suggested that green diseased pseudostems only show sparse growth of hyphae in discolored vascular strands, and sporulation is sparse or absent. It was also suggested that the abundant sap exuding from the cut end of such pseudostems is free from conidia but contains fragments of hyphae. There is the need to re-assess the risk sap poses during the detection and destruction of an infected plant. Nevertheless, cane knives should be cleaned and sterilized between plants. In their sap studies, Rishbeth (1955) and Stover (1954, 1956) did not differentiate between the laticifer, phloem and xylem fluids. The initial sap that exudes from a cut pseudostem comes from laticifers. The osmotic potential of the contents of the laticifer is lower (more negative) than the surrounding tissues, so they have a positive turgor. The contents of the laticifer exude when cut because the cutting sets the turgor pressure of the laticifer to zero, and then the osmotic potential gradient between the latex and surrounding cells causes water to flow into the laticifer along its length. This causes the sap to flow out of the cut surface of the pseudostem (**Figure 9**). Sap stops flowing when the osmotic gradient becomes zero. Once the laticifers empty, the xylem will start to exude fluid because of root pressure. Laticifer sap is quite milky whereas xylem fluid is quite clear. Foc does not grow in a healthy laticifer. If it was infected it would lose turgor. It is the xylem vessels that harbor the pathogen.

– There is considerable potential for insects to spread conidia from plant to plant. Meldrum et al. (2013) found the pathogen on exoskeletons of the banana weevil borer, *C. sordidus*, and suggested that it may be a vector.
– There is no evidence of dispersal in fruit, even when the bunch stalk is infected. Fruit from seriously infected plants rarely produce a marketable bunch. However, the Australian Government considered that the pathogen could move as symptomless infections of the vasculature of fruit crowns (DAFF, 2004).
– Sporodochia (macroconidia bearing hyphal masses) could provide local dispersal of TR4 directly by rain splash, or by being washed into the soil. Although these structures have been observed on the stems of infected banana plants in a glasshouse, their role in the field needs to be examined (Ploetz, 2015).

## Disease Spread

This section is concerned with the spread of the disease as distinct from the dispersal of the pathogen. It involves the banana plant and its reaction to the pathogen and the environment.

An epidemic of the disease usually begins with one or a few affected plants. The spread from one plant to another takes place essentially by overlapping roots of interconnected mats (active spread), whereas new centers of infection result from the passive dispersal of inoculum. Stover and Waite (1954) suggested that high inoculum levels were necessary for rapid disease development as this increased the number of roots that were infected, and the possibility that the pathogen would enter the rhizome. Intensive cultivation, vehicle movement, failure by farm staff to apply basic farm hygiene procedures and (perhaps most importantly) frequent irrigation from a contaminated water source, will greatly facilitate the development of new foci and accelerate the epidemic.

Stover (1962) noted that the disease spread more slowly on small, compared to large, plantations of ‘Gros Michel.’ He attributed this to small farms being separated by banana-free blocks of land that slowed disease spread. Also, small farms may rely on rainfall and not have irrigation water which can spread the pathogen.

When populations of the pathogen are low in soil, new foci usually appear as single or paired plants, often some distance from previous centers of disease (Stover, 1962; Deacon, 1984). There may also be a long incubation period before symptoms appear. However, as the epidemic progresses and inoculum increases, symptoms develop more rapidly and clumps of six to twenty or more plants are affected in a random pattern (Stover, 1962). Fawcett (1913) also noted that when inoculum levels were low, only isolated cases of the disease were found and external symptoms in such plants were not expressed until after bunch initiation. He also reported that there was ‘dwarfing or stunting’ of plants in fields where the disease had been present for some time and inoculum levels had increased.

The fungus can cause disease at very low initial inoculum levels (Nash-Smith, 1970). Since the banana plant remains in the soil for many years (Stover and Waite, 1954) and the roots search through a large volume of soil, the probability that these roots will contact inoculum present in the soil is high. Thus, limiting inoculum production is an important management goal.

## Survival

TR4 is a soil-borne fungus that is well adapted to long-term survival in soil. It readily forms chlamydospores, which remain dormant in the remnants of decayed host tissue until stimulated to germinate by root exudates from banana, alternative hosts or pieces of fresh plant remains (Stover, 1962). It is virtually impossible to eliminate from infested soil by crop rotation and even by bare fallowing. Stover and Waite (1954) mention a survival figure of 40 years for Foc in abandoned fields but there are many suggestions that it may be much longer.

**Figure 10:**
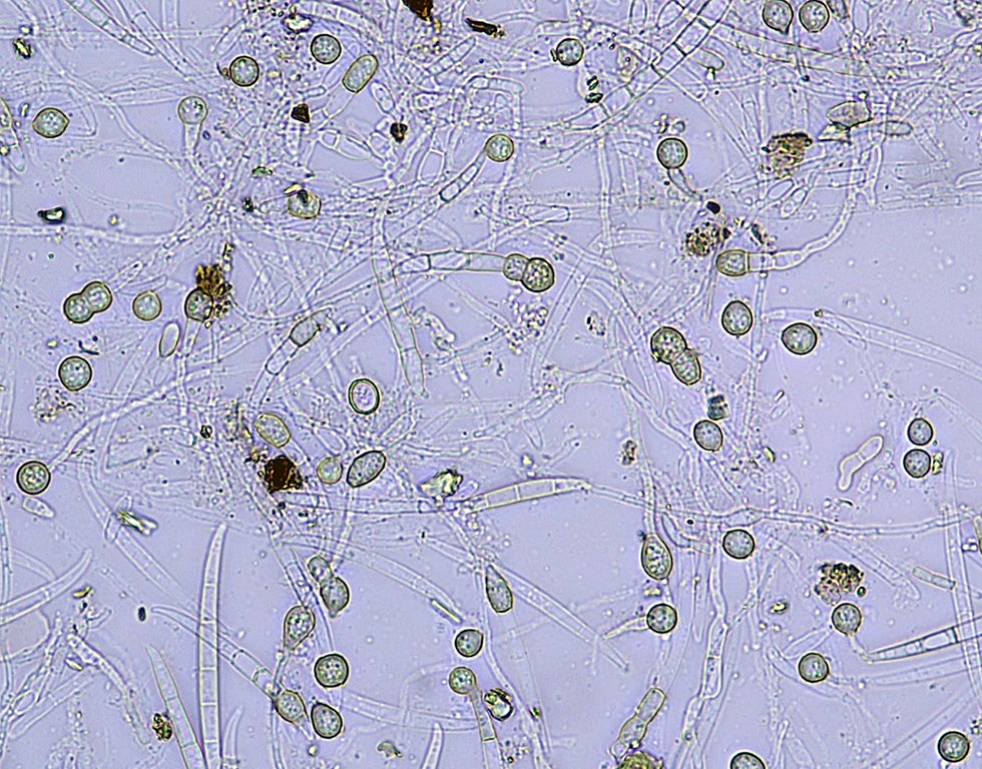
Chlamydospore production in a Foc culture (W. O’Neill).

Chlamydospores produced by Foc in dead and dying banana plants are released into the soil when the plant material decays (**Figure 10**). They are undoubtedly the most important survival propagule of the pathogen, but it is difficult to determine how long they survive naturally in the soil. Chlamydospores can persist for an extended period in plant debris in soil in the absence of a suitable host plant.

When examining infested banana soil, Trujillo and Snyder (1963) found chlamydospores associated with the cortical tissue of decayed banana roots, but could not find them in the decayed tissue of other plants. Their presence in soil without plant tissue was difficult to ascertain. They found them to be very sparse and unevenly distributed in the soil and suggested that chlamydospores in plant tissue survive longer than those unprotected in the soil. Chlamydospores of the Fusarium wilt pathogen of date palm (*Fusarium oxysporum* f. sp. *albedinis* Gordon) are known to persist in soil at a depth of 1 m (Louvet and Toutain, 1981). It is thought that chlamydospores of Foc are probably found at a similar depth in the soil to which the primary roots can penetrate.

Stover (1962) suggested the long survival in soil is indicative of multiplication on wild hosts or to its saprophytic ability, and suggested it was a soil saprophyte with pathogenic potential. However it was not a frequent saprophytic colonizer of dead banana tissue in soil. This suggests that chlamydospores in plant tissue in the soil originated from a living plant. Early studies do not indicate how these propagules survived; whether in a dormant state or by germination and formation of new chlamydospores when activated by nutrients. Macro- and microconidia are found on the surface of dead plants, but are very fragile and die quickly when exposed to sunlight (Manicom, 1989).

Better understandings are needed for the formation, germination and survival of chlamydospores, as it could inform the containment of TR4. Nonetheless, the ability of the pathogen to colonize non-susceptible weeds and grasses complicates containment (see the section *Alternative Hosts* below). It can be seen from this review that the majority of studies on Foc survival in soil are more than 50 years old. This brings into sharp relief the critical need for more research on survival of the pathogen and inoculum management, especially given the continuing spread of TR4 epidemics.

To summarize, these studies indicate that the pathogen in soil originates in tissues of diseased banana plants which were colonized while the plant was still alive. The highest populations of the fungus occurred around a diseased plant, and they declined significantly once affected plants were removed (Stover, 1962). This illustrates the importance of the current destruction protocol for TR4, which is designed to reduce the amount of inoculum reaching the soil. Early detection is essential; once the vascular pathogen reaches the leaf lamina, the fungus is no longer confined to the xylem; it will move into the phloem and parenchyma and produce chlamydospores and microconidia (Wardlaw, 1961).

Flood fallowing was used extensively at the end of the ‘Gros Michel’ era where production occurred in alluvial flood plains (where flooding was possible). In general, pathogen survival was minimized in saturated soils after 6 weeks (Stover, 1954). However, since flooding reduced the populations of most soil organisms, recontamination of the resulting biological vacuum by the pathogen was a routine occurrence (Stover, 1962). Crop rotation with paddy rice was considered in Taiwan. Su et al. (1986) found that in submerged soils, the population of Foc dropped to a non-detectable level within 4 months. Field tests showed that rotation with paddy rice for 1 and 3 years reduced the disease incidence from 40 to 12.7% after 1 year and 3.6% after 3 years. Disease incidence was not decreased when infested banana fields were rotated with alternate crops such as sugarcane or sunflowers for 3 years. Crop rotation with a perennial pineapple crop in TR4 infested soil in Northern Territory, Australia, allows the land to be re-planted with banana to produce two cycles (plant crop and first ratoon). Readers who are interested in crop rotation should refer to Dita et al. (2018).

## Alternative Hosts

At an international symposium on soil-borne plant pathogens in 1963, S. D. Garret (1970) stated that despite the great volume of work done on the non-host phase of Foc, “it is still uncertain which is the most important among several modes of survival in the non-host phase.” This statement is still applicable.

Chlamydospores of Foc in dead plant material are generally accepted as the major means of its survival, but its persistence may be primarily due to its capacity to colonize the outer root cells of the epidermis and cortex of grasses and weeds as asymptomatic endophytes (Ploetz, 2015). Armstrong and Armstrong (1948) first showed that vascular wilt Fusaria were able to parasitize the roots of plants without pathogenesis.

Grass and weed populations may serve as a reservoir of inoculum that influences the occurrence of disease in banana plantations. They are also possibly involved in the contamination of irrigation sources and rivers with the pathogen. However, alternative hosts are usually regarded as a mechanism of long-term survival and not for the build-up of the fungus in the soil. A better understanding is needed of the importance of alternative hosts when managing TR4.

Waite and Dunlap (1953) isolated Foc (presumably race 1) from alternative grass and weed hosts. They collected grass and weed species from a field in Honduras with a high incidence of Fusarium wilt, planted them in a sterile sandy loam and inoculated them with Foc grown in corn meal sand. Of many plants tested, three species of grass [*Paspalum fasciculatum* Willd., *Panicum purpurascens* Raddi, *Ixophorus unisetus* (Presl.) Schlecht.], and one herb (*Commelina diffusa*) were infected. They also recovered Foc from the roots of these plants in the field.

In Taiwan, Su et al. (1986) examined 25 species of weed and eight rotation crops growing in a race 4-infested field. Three sedges (*Cyperus iria* L., *Cyperus rotundus* L., *Fimbristylis koidzumiana* Ohwi) and one herb (*Gnaphalium purpureum* L.) were infected, and they confirmed the pathogenicity of these isolates on Cavendish.

In Australia, Pittaway et al. (1999) reported that STR4 (VCG 0120) was isolated from surface sterilized roots of a grass (*Paspalum* sp.) and a weed (*Amaranthus* sp.) in soil adjacent to Cavendish plants affected by STR4 in southern Queensland. In 2005, 18 species were collected from within a 1-m radius of Cavendish bananas affected by TR4 in the Northern Territory (Hennessy et al., 2005). TR4 (VCG 01213) was isolated from four species: *Chloris inflata* (a common grass), *Euphorbia heterophylla* (Euphorbiaceae), and *Cyanthilium cinereum* and *Tridax procumbens* (Asteraceae).

The presence of Foc as asymptomatic endophytes in weeds and grasses calls into question the value of current containment protocols for Fusarium wilt where the focus is on killing infected banana plants and asymptomatic hosts are largely ignored. Their importance as reservoirs for the pathogen and as potential vehicles of spread should be examined under natural conditions. Likewise, whether their removal would be helpful also requires study.

Since most alternative host studies have been conducted either in glasshouse trials or in heavily infested field soils, these results may well overstate the capacity of the plant species to be an alternative host under natural field conditions. Based on the old literature, it is clear that Foc is capable of surviving at very low levels which, nonetheless, provide sufficient inoculum to start epidemic disease development. With modern techniques it should be possible to follow the survival of TR4 in soil in the absence of a banana host and understand the importance of asymptomatic weed infection and/or chlamydospore survival in perpetuating this pathogen.

## Urea to Reduce Populations of *Fusarium oxysporum* f. sp. *Cubense* in Soils

Urea, when incorporated in soil, reduces the populations of certain soil-borne fungi by the production of volatile ammonia upon hydrolysis (Tsao and Oster, 1981; Chang and Chang, 1999). In the containment of TR4 in North Queensland, urea is utilized to reduce the soil population of the fungus. Regular surveillance results in the early detection of wilt symptoms. Once the pathogen is confirmed, affected plants are injected with herbicide and a high rate of urea (1 kg per square meter) is applied to the soil surface surrounding the diseased plants to reduce propagules of the fungus in the soil. The area is then covered with plastic sheeting to produce a lethal concentration of ammonia. There is currently no systemic fungicide available for killing the pathogen in an affected pseudostem. As the pseudostem and leaves are a potent source of inoculum, they are chopped up and placed in large plastic bags and 1 kg of urea is added to each bag (Persley et al., 1989). When the urea treatment was used previously to contain an outbreak of STR4 in South-east Queensland and northern New South Wales, symptomatic plants were not injected with herbicide prior to bagging. This was because killing by herbicide is thought to predispose plants to rapid fungal colonization with a greater production of inoculum in the affected plant. For this reason, the use of herbicide in the current TR4 destruction protocol is being reviewed.

Urease, which plays a vital role in overall nitrogen metabolism and catalyzes the hydrolysis of urea to give volatile ammonia, occurs in many bacteria, yeasts and higher plants. Production in fungi is variable (Veverka et al., 2007). Foc contains urease (Sequeira, 1963) and it is also present in the growing point and floral shoot apex of the banana plant (Freiberg and Payne, 1957) and these sources likely catalyze the urea to produce ammonia when the infected plant material is bagged.

Although volatile ammonia produced from the hydrolysis of urea causes a rapid decline in the population of *Fusarium*, nitrite accumulation is considered to provide an additional longer term source of fungicidal action where ammonia levels have been sufficiently high to inhibit the activity of *Nitrobacter*, which converts nitrite to nitrate (Sequeira, 1963; Smiley et al., 1970; Loffler et al., 1986).

The urea treatment would be expected to kill Foc present in infected banana roots. Chang and Chang (1999) found that 3,000 mg kg^-1^ of urea incorporated in soil to give 400 mg kg^-1^ ammonia completely killed *Phellinus noxius*, which causes brown root disease in numerous orchard and forest tree species, in wood pieces less than 3 cm in diameter.

Ammonia is reported to be more effective in soils with relatively low soil moisture (–0.75 to –0.15 MPa) than in soils with high soil moisture (–0.025 MPa) (Chang and Chang, 1999). In wet soils the ammonia dissolves in water to form the ammonium ion and would not be present as a fumigant. Ammonium/ammonia and nitrite/nitrous acid each exist in equilibrium in aqueous solution. The non-ionized form of each compound penetrates cell membranes more easily and is thus more toxic to Foc than the ionized form (Tsao and Oster, 1981). The ammonium ion generated in moist soils may eventually stimulate the activity of an antagonistic microflora which further reduces the survival of Foc.

Many nitrogenous amendments are phytotoxic when used in high concentrations. When urea is applied at a high rate, ammonium toxicity and alkalinity, along with nitrite accumulation, will damage plants. Crop safety is not an issue when using urea to treat soil in the vicinity of an infected banana plant. It may even have a positive effect in that soil toxicity will be detrimental to the health of alternative weed hosts.

## Fungicides to Reduce Inoculum Production in Plants During the Destruction Process

While fungicides have not been effective for managing Fusarium wilt in the field, they could reduce inoculum when infected plants are destroyed in an eradication or containment program. Injection of pseudostems with herbicides is a common strategy for destroying infected and associated plants; however, dead and dying plants could continue to be a potent source of inoculum. During the death of an infected plant, there is a high likelihood that the fungus will move from the colonized xylem vessels into the surrounding parenchyma and cortical tissues, where it would undoubtedly produce many microconidia and chlamydospores unless the infected plant material is treated in some way. In Cavendish and ‘Lady Finger’ plants inoculated with GFP-labelled STR4, Warman and Aitken (2018) demonstrated the production of sporodochia, hyphae and chlamydospores on the outer surface of senescing leaf sheaths, as well as chlamydospores in the gas spaces of senescing leaf sheaths. This result suggests that the use of herbicides is likely to encourage rapid colonization of senescing plant material, thus increasing inoculum potential.

Injection of infected pseudostems with an appropriate fungicide at the time of herbicide injection could provide some reduction in fungal sporulation, although which fungicides would do so is unclear. In India, injection of rhizomes with carbendazim (3 ml of a 2% suspension) was reported to protect ‘Rasthali’ (AAB, ‘Silk’ subgroup) bananas against the disease (Lakshmanan et al., 1987), but this was done within the context of disease management rather than plant destruction/inoculum reduction. While rhizome injection with carbendazim in association with an herbicide would likely reduce inoculum production in dead and dying banana plant tissue, its use, or the use of other fungicides, may raise health and safety concerns for those applying the treatments. Other fungicides may be appropriate for this purpose but require further evaluation. Nel et al. (2007) reported that prochloraz and propiconazole significantly inhibited *in vitro* mycelial growth of STR4, with prochloraz giving complete inhibition at all concentrations tested (1–100 µg ml^−1^). Prochloraz and propiconazole were also highly effective when applied as a root dip to ‘Chinese Cavendish’ plants inoculated with STR4, reducing disease severity by 80% and 75% respectively compared with control plants. From a health and safety perspective, these fungicides may be more appropriate alternatives to the benzimidazole fungicides such as carbendazim and benomyl, although studies are required to establish their ability to reduce sporulation of Foc in infected banana tissue treated with herbicides, as well as their mobility in infected plant tissue following injection.

## TR4 Control and Containment in Queensland, Australia

Following the confirmation of a TR4 incursion in Queensland in 2015, a cooperative effort between the State Government and the banana industry resulted in the rapid containment of affected sites, implementation of best-bet control strategies and accelerated research on management options. This program has resulted in minimal spread of the disease, with only three properties affected more than four years later. The key aspects of this program have been:

- An intensive surveillance and diagnostics program for early detection of diseased plants.
- Destruction of symptomatic and surrounding healthy plants at disease foci (including the use of urea, as detailed in the section Urea to Reduce Populations of *Fusarium oxysporum* f. sp. *cubense* in Soils), decontamination of all equipment and personnel involved in the destruction process, and restriction of further entry into affected areas.
- Implementation of on-farm biosecurity practices and hygiene, including farm zoning to restrict movements onto (and within) banana farms, and the use of disinfectants in footbaths and for vehicle and machinery decontamination.
- Use of clean (tissue cultured) planting material.

Nguyen et al. (2019) found that quaternary ammonium compounds containing a minimum of 10% active ingredient were the most effective against race 1 and TR4. When used at a 1:100 dilution, the survival of all Foc propagules was completely inhibited, regardless of the absence or presence of soil (at 0.05 g ml^-1^). Further resources are available online *via* the Australian Banana Growers Council and Biosecurity Queensland websites^1^.

## The Epidemiology Approach to Containment

Epidemiology (derived from the Greek epi = on, demos = population) is concerned with disease on populations of plants; it recognizes the influence of the environment, and the importance of time in disease development (MacKenzie et al., 1983; Brown et al., 1997). Quantitative epidemiology is all about numbers, and for Fusarium wilt might consider the number of pathogen propagules that reside in soil or infected plants, the number of successful infections that enter the rhizome, the ratio of diseased to healthy plants, the number of days for disease latency (inoculum generation period) and incubation (infection to symptom expression period), and the number of days that an epidemic lasts.

The epidemiological implications of the source of the initial inoculum, the means of pathogen dispersal, sanitation strategies, host range of the pathogen, environmental conditions for disease development, latency, and time required to manage the epidemic, are all important when managing containment.

**Figure 11:**
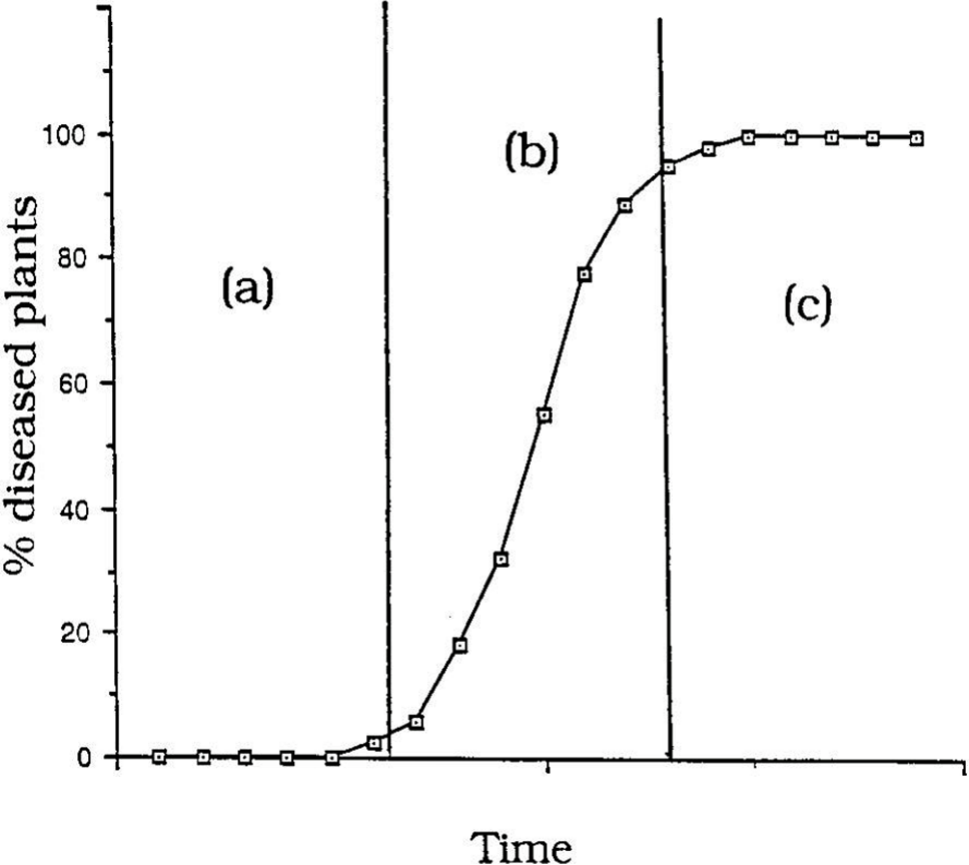
‘Compound interest’ disease epidemic showing a sigmoidal (S-shaped) epidemic progress curve: (a) initial lag phase, (b) exponential phase, (c) decline phase (from Brown et al., 1997).

As bananas are a perennial crop and Foc inoculum carries over from the previous season and increases during the current season, Fusarium wilt is regarded as a polycyclic or ‘compound interest’ disease. The pattern of the epidemic, in terms of the number of symptomatic plants, is given by a curve called the disease-progress curve, which shows the progress of the epidemic over time. A sigmoid curve (S-shaped) is characteristic for polycyclic diseases (**Figure 11**). The curve has three phases:

- initial lag phase where inoculum levels are low.
- exponential or logarithmic phase where inoculum levels and plants available for infection are not limiting.
- decline (plateau) phase where very few plants are available for infection.

With compound interest diseases new inoculum produced (= interest) is continuously added to the previous amount of inoculum (= interest bearing capital). The epidemic will start slowly (lag phase) but accelerate rapidly in the exponential phase, often with catastrophic losses.

A major focus for the TR4 epidemic in Queensland is to keep the amount of inoculum available for infection as low as possible to reduce the rate of disease spread, i.e. keep the disease in the lag phase. Once a diseased plant is detected, it and surrounding plants are killed with herbicide before inoculum is released into the soil. This will eliminate the disease but inoculum of the pathogen in the plant is not killed. Therefore plants are cut down, sealed in bags and surrounding soil treated with urea before covering with plastic. Plants are not dug out in the current strategy as this causes soil disturbance and possible spread of infested soil during flooding rains. Rhizomes are gouged out and urea applied to the cavity to hasten decomposition and reduce inoculum levels. At the time of writing, the most effective way of treating infected plants is being investigated (see the section Urea to Reduce Populations of *Fusarium oxysporum* f. sp. *cubense* in Soils).

## Case Study: the Development of the TR4 Epidemic in Taiwan

The progress of the TR4 epidemic in Taiwan was first documented by Su et al. (1986). Commercial banana production in Taiwan involves many small farms (0.5–1.0 ha) rather than the extensive plantations managed by multi-national companies in the more tropical regions of the world. Taiwan grew Cavendish for export to Japan.

Since TR4 is virtually impossible to eradicate, their attempt to contain the disease was originally aimed at reducing the soil population of the fungus. Their disease management strategy was to dig out diseased plants, cut them into pieces, treat the pieces with lime and bury them 60 cm or deeper in the soil. The surrounding eight ‘healthy’ plants were destroyed in the same way. However this strategy proved difficult and failed to provide long term control. As the epidemic progressed, growers became careless, did not bury infected material, but left it lying on the ground or threw infected pseudostems into irrigation channels.

The first diseased Cavendish plant in Taiwan was detected in 1967. By 1976 there were half a million infected plants spread over 1,200 ha (Su et al., 1986). In 1983, 1,500 ha were infested (Hwang and Ko, 2004) and in 1999, 3,000 ha were infested (Hwang, 2001).

**Figure 12:**
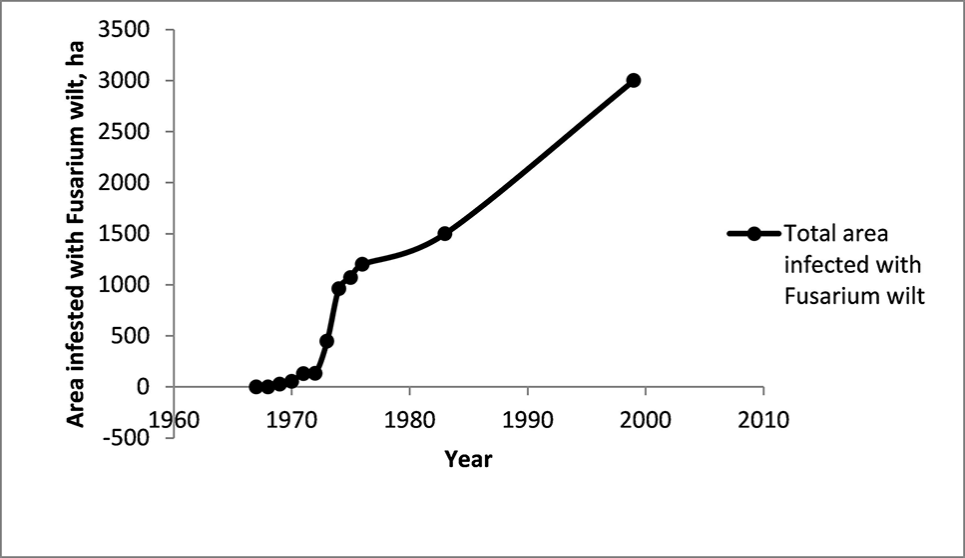
The area infested with Fusarium wilt in the Cavendish banana industry in Taiwan. Data from Su et al. (1986), Hwang (2001), Hwang and Ko (2004). The industry covered 50,000 ha in the mid-1960s and was 6,000 ha in 1999.

The following graph illustrates the progress of the TR4 epidemic in Taiwan (1967–1999) (**Figure 12**).

Over more than 30 years the total area infested has increased, with some important changes in the rate of spread of the disease. Initially, at least on an absolute basis, the spread appeared slow, but increased very rapidly in the mid-1970s (**Figure 12**). After this there seems to have been a steady increase in the area infested. Rigid control measures introduced in 1970 were abandoned in 1973 because they were unpopular, difficult to apply and had not prevented the spread of the disease.

When data for the spread of the disease is expressed as the number of hectares newly infested each year (ha/y), a rapid expansion of the disease in Taiwan in the mid-1970s is clearly evident. This illustrates the need for early intervention if the disease is to be contained.

**Figure 13:**
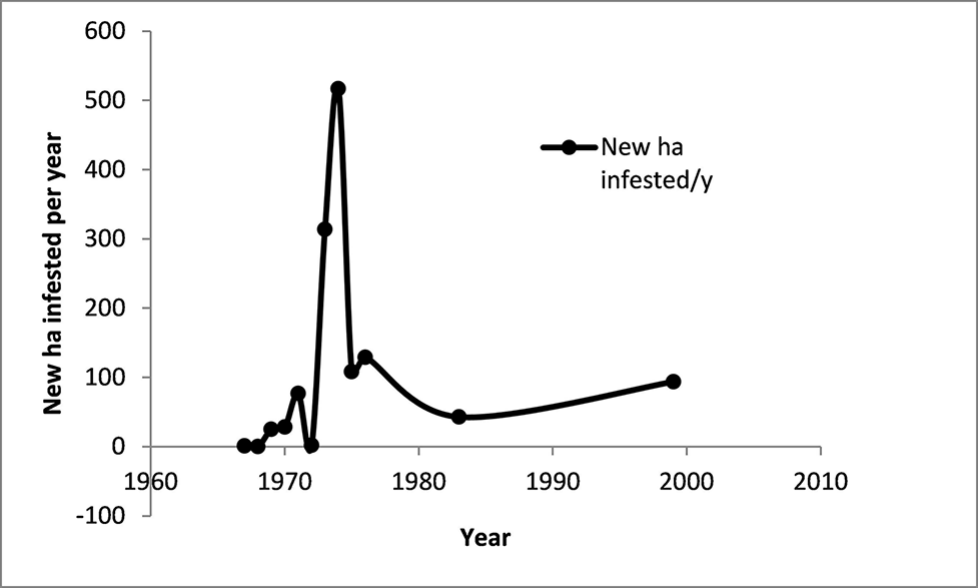
The annual rate of spread of Fusarium wilt in the Cavendish banana industry in Taiwan. Data derived from Su et al. (1986), Hwang (2001), Hwang and Ko (2004).

Calculations from the data from Taiwan show the rapid increase in the rate of spread of the disease in the early years of the epidemic (**Figure 13**). This demonstrates the need for urgent action when an outbreak is first detected.

The origin of inoculum for the initial TR4 outbreak in North Queensland is unknown. Based on the number of plants infected, and the long incubation period of the disease, it is possible that the disease was present for some time before it was detected. However, there was very limited secondary spread on the infested property from the initial incursion prior to the banana farm being taken out of production. TR4 has subsequently been found on another two properties.

## Conclusions

Despite the long history of Fusarium wilt in Australia (145 years), the only effective way of managing the disease is still by exclusion or early containment. To safeguard existing Cavendish plantations until acceptable resistant cultivars become available, a better understanding of the epidemiology and pathology of TR4 is required. The greatest challenge will be to confine the fungus to an existing area and to prevent the dispersal of its propagules. This will depend on focused research to provide information on how to improve early detection and destruction of infected plants and intensive treatment of soil in their vicinity. Research into better methods for detecting the presence of the pathogen in soil and water is also required.

The only reliable way of measuring the progress of the epidemic is the symptomatic plant. With any plant disease there is an incubation period during which symptoms are not evident. The susceptibility or resistance of the host plant, as well as environmental factors, influence incubation period. The shorter the incubation period, the more rapidly pathogen populations increase. Soil populations of the pathogen are extremely important. A high population will increase disease incidence and severity as well as the potential for disease spread. It will result in an earlier development of wilt symptoms and plants will die more quickly. Low soil populations will give systemic invasion, but plants will generally take longer to produce recognizable symptoms. Disease development is also strikingly influenced by both environmental and host factors, and disease incidence and severity may fluctuate independently of the population level. For example a saturated soil, which reduces the oxygen concentration in the roots, will make the plant more susceptible to infection. Once symptoms are detected, early intervention is possible. However, the absence of symptoms does not necessarily indicate that the pathogen is not present. It is possible for a very low population of the fungus to be present in a banana field where plants do not show any wilt symptoms. It may take as long as five years for wilt symptoms to appear in a field with a very low population (Rishbeth and Naylor, 1957). Thus once the pathogen enters a growing area, long-term surveillance will be required.

It is important to clarify the role of sap from laticifers and xylem fluid in the dispersal of the pathogen. It is generally accepted that a large number of microconidia are extruded in xylem fluid and thus it is a potent source of inoculum for pathogen spread. Testing using traditional and molecular (fluorescence microscopy and qPCR) methods is required to track the movement of the pathogen in the vascular system of banana. As the disease can have a long incubation period, the rate of colonization may not preclude spread solely by hyphal growth.

Another important factor to consider is growing banana somaclones which are not completely resistant to TR4 when containment is being attempted, and the disease has not become widespread. There are Cavendish somaclones available from Taiwan, China, Australia and Indonesia which have a measure of resistance to Foc. However, resistance is not complete and growing such plants becomes an epidemiological risk, especially in regions where the pathogen has a limited distribution, as they may mask its presence and spread. It may be prudent to delay the deployment of such cultivars until containment strategies have failed and the disease is widespread. The current approach is to grow these somaclones using an integrated approach to managing Fusarium wilt. The underlying theme of this program is the promotion of practices to enhance ‘soil health’ as well as the management of plant stress. Such an approach needs to consider temporal variations in host susceptibility and pathogen activity. There are a number of environmental factors which will affect both; especially physical environmental factors in the soil (temperature, moisture, aeration). Many banana growing areas are subject to periodic cyclones and tropical storms where the best drained soils become temporarily saturated and overland flow of water occurs. A water-saturated, oxygen deficient soil will increase plant susceptibility, and partially resistant plants will succumb. Thus, extreme environmental events may quickly override any gains made in disease suppression through integrated management.

Despite current containment and surveillance protocols, confinement is likely to fail where there has been a long time period between the introduction of the pathogen and its detection, along with increased mechanization in the modern industry, high annual rainfall and flooding in tropical storms, and failure to apply simple farm hygiene procedures. One also must be realistic and recognize that the inoculum level in a TR4-infested soil will never be zero, and that these soils will remain a source of inoculum for decades and not sustain economic banana production as we know it today unless an acceptable resistant cultivar becomes available.

Although the entire banana industry is at risk from TR4, even if the pathogen is not contained, the disease is unlikely to spread as rapidly as it has in the export plantations in Asia and Mozambique. During the ‘Gros Michel’ epidemic the disease spread more slowly on small farms than in large plantations. Banana farms in Queensland are quite dispersed, growers are very progressive, and most have implemented on-farm biosecurity measures.

## Author’s Note

**Figure 14:**
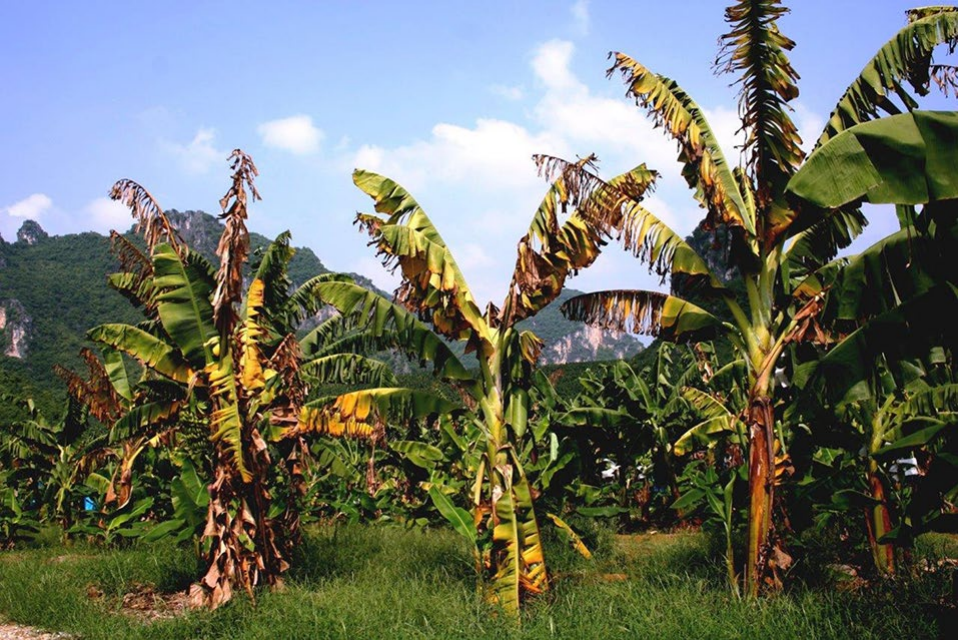
Cavendish banana plants affected by Fusarium wilt tropical race 4, showing yellowing and collapse of leaves at the petiole base (John E. Thomas).

The aspects of Fusarium wilt of banana discussed in this review are generally related to race 1 and subtropical race 4 of *Fusarium oxysporum* f. sp. *cubense*, but are considered by the authors to be important to understanding the epidemiology and pathology of tropical race 4 (**Figure 14**). The basic biology of the fungus should be considered when evaluating surveillance and containment options and quarantine protocols for the disease.

## Author Contributions

KP and LC reviewed the literature and wrote the main body of the article. WO’N had primary input into the sections on the influence of nematodes, alternative hosts, urea soil applications and TR4 containment, and supplied many images. DT prepared the section on the Taiwan TR4 case study and had significant input into the sections on influence of climatic factors and pathogen spread. All authors contributed to manuscript revision and editing.

## Funding

Funding for this review was provided by Biosecurity Queensland and Agri-Science Queensland (Department of Agriculture and Fisheries).

## Conflict of Interest

The authors declare that the research was conducted in the absence of any commercial or financial relationships that could be construed as a potential conflict of interest.

## Footnotes

1 https://abgc.org.au/panama-tropical-race-4/ https://publications.qld.gov.au/dataset/panama-disease-tropical-race-4-grower-kit
